# Machine learning applications across living environments, activity behaviors, and mental health among older adults: a systematic review

**DOI:** 10.3389/fpubh.2026.1942765

**Published:** 2026-10-26

**Authors:** Yuxuan Wang, Jun Lan

**Affiliations:** School of Human Settlements Science, University of the Chinese Academy of Sciences, Beijing, China

**Keywords:** older adults, living environment, activity behaviors, mental health, machine learning, systematic review

## Abstract

**Background:**

Machine learning is increasingly used to examine how living environments, activity behaviors, and mental health are interconnected in later life. However, evidence across these domains remains dispersed, and the contribution of machine learning to understanding their relationships has not been systematically synthesized.

**Methods:**

Following PRISMA 2020-informed procedures, we systematically reviewed empirical studies published between 1 January 2016 and 30 August 2026. Eligible studies included participants aged 60 years or older or reported separately extractable findings for this age group, examined relationships involving at least two of living environment, activity behaviors, and mental health, and used machine learning as a substantive component of the empirical analysis.

**Results:**

Of 4,337 records identified, 27 studies met the eligibility criteria. Evidence was concentrated on individual relationships between living environment, activity behaviors, and mental health, while relatively few studies considered all three domains together. Across this literature, machine learning was used to construct environmental and behavioral measures, predict behavioral and mental-health outcomes, and characterize nonlinear relationships, thresholds, interactions, and population or spatial heterogeneity. Collectively, these studies point to considerable variation in environment–behavior–health relationships across populations, places, and analytical contexts. Nevertheless, the evidence remains distributed across partially connected relationships, with limited integration of activity behaviors into analyses linking living environments with mental health.

**Conclusion:**

Current research provides a growing but fragmented evidence base on the interconnections among living environments, activity behaviors, and mental health in later life. Machine learning offers new opportunities to characterize the complexity and heterogeneity of these relationships. Greater integration of environmental, behavioral, and mental-health evidence across populations, places, and time could provide a stronger basis for understanding how living environments relate to mental health in later life.

## 1 Introduction

Population aging is reshaping public health, urban development, and the provision of supportive environments for later life (1, 2). Healthy aging is influenced not only by individual characteristics but also by the environments in which older adults live and conduct their daily activities (1, 2). This review defines the living environment (LE) as the physical and natural conditions within older people’s homes and their surrounding neighborhoods (2), which can be summarized into four dimensions: housing and living conditions, including floor area, the condition of indoor flooring, and infrastructure such as water, electricity and gas (3); the built environment, including population density, land-use patterns, road networks and the morphology of street spaces (4, 5); the natural environment, comprising parks and green spaces, vegetation cover, visible greenery in the streetscape, and ‘blue spaces’ such as rivers and lakes (5–7); and environmental quality, encompassing exposure factors such as air pollution and environmental noise (7, 8). More supportive residential and neighborhood conditions, including better-maintained housing, greater access to green and blue spaces, more favorable street conditions, and better provision of public facilities, have generally been associated with higher physical activity and better mental well-being among older adults (2, 9–13). However, the direction and magnitude of these associations may vary according to the environmental attribute, population subgroup, and outcome examined. Understanding how older adults interact with their living environments therefore requires consideration of both environmental characteristics and the activity behaviors through which these environments are experienced and used.

Activity behaviors (AB) refer to the observable or self-reported ways in which older adults act within, move through, and use their living environments. Previous research has linked built and neighborhood environments to physical activity, walking, and active aging (12, 14, 15), while more recent studies have examined walking, cycling, public-transport use, driving, and other forms of daily mobility among older adults (4, 16–23). In this review, AB broadly encompasses physical activity, mobility, social activity, and public-space activity/use, capturing multiple ways in which older adults interact with and move through their everyday environments. Walking and cycling may be classified as physical activity behaviors or mobility behaviors depending on how they are operationalized in individual studies. Detailed coding criteria are provided in Section 2.3.

Different forms of activity behavior have shown distinct associations with mental health in later life. Social participation and volunteering have been associated with fewer depressive symptoms (24, 25), while greater social participation has been linked to better psychological health (26), higher life satisfaction (27), and higher subjective well-being (28). A later cohort study found that greater social participation was associated with fewer depressive symptoms, although the magnitude of this association varied across population subgroups (29). Exploratory sensor-based findings suggested that greater mobility was associated with less loneliness (30); older adults with depressive symptoms also showed lower measured activity levels (31) and slower walking speed (32). Living environments (LE), activity behaviors (AB), and mental health (MH) can therefore be considered interconnected domains of healthy aging.

The growing availability of diverse data sources has expanded the methodological possibilities for investigating these relationships, including spatial data (e.g., GIS-based neighborhood characteristics), image data (e.g., street-view and remote-sensing imagery), mobility data (e.g., GPS trajectories and travel records), sensor data (e.g., accelerometer and wearable-device measurements), and health data (e.g., survey-based mental-health and health-status measures). Recent studies increasingly integrate these data sources to characterize environmental exposures, daily activities, and health outcomes among older adults (33–37). Machine learning (ML) offers analytical tools for extracting information from complex and high-dimensional data, modeling nonlinear relationships and interactions, and supporting prediction and interpretation (33, 38, 39). These capabilities have made ML increasingly relevant to research at the intersection of environmental, behavioral, and mental-health dimensions of aging.

Despite this methodological development, existing reviews have largely synthesized separate components of the field (13). Reviews of neighborhood and built environments have examined relationships with older adults’ health, physical activity, walking, well-being, and social participation (11–13), while other reviews have focused on social isolation, loneliness, or community participation in later life (40–43). Environmental mental-health research has also examined potential pathways linking environmental exposures with mental-health outcomes (44), whereas methodological reviews of street-view imagery, urban analytics, big data, and ML have primarily focused on environmental measurement, data sources, or broader health applications (33–36, 38, 45). A limitation of the existing review literature is that the application of ML across the partially overlapping relationships among LE, AB, and MH in older adults remains insufficiently synthesized.

An important unresolved issue is that ML can serve different analytical functions across this literature, and these functions address different research questions and provide different forms of evidence. Distinguishing how ML contributes to measurement, modeling, interpretation, and inference is therefore important for understanding both its methodological contribution and the evidential scope of current research.

Accordingly, guided by the analytical framework shown in Figure 1, this systematic review synthesizes empirical studies applying machine learning across living environments, activity behaviors, and mental health among older adults. It examines the partially overlapping LE–AB, AB–MH, LE–MH, and LE–AB–MH relationships represented in the literature and addresses three questions: (1) What functional roles does ML perform across studies of LE, AB, and MH among older adults, and which methods are used for these purposes? (2) How does ML contribute to the measurement, modeling, interpretation, and inference of relationships across these domains? (3) What methodological strengths, limitations, and evidence gaps characterize current ML applications in this field?

**Figure 1:**
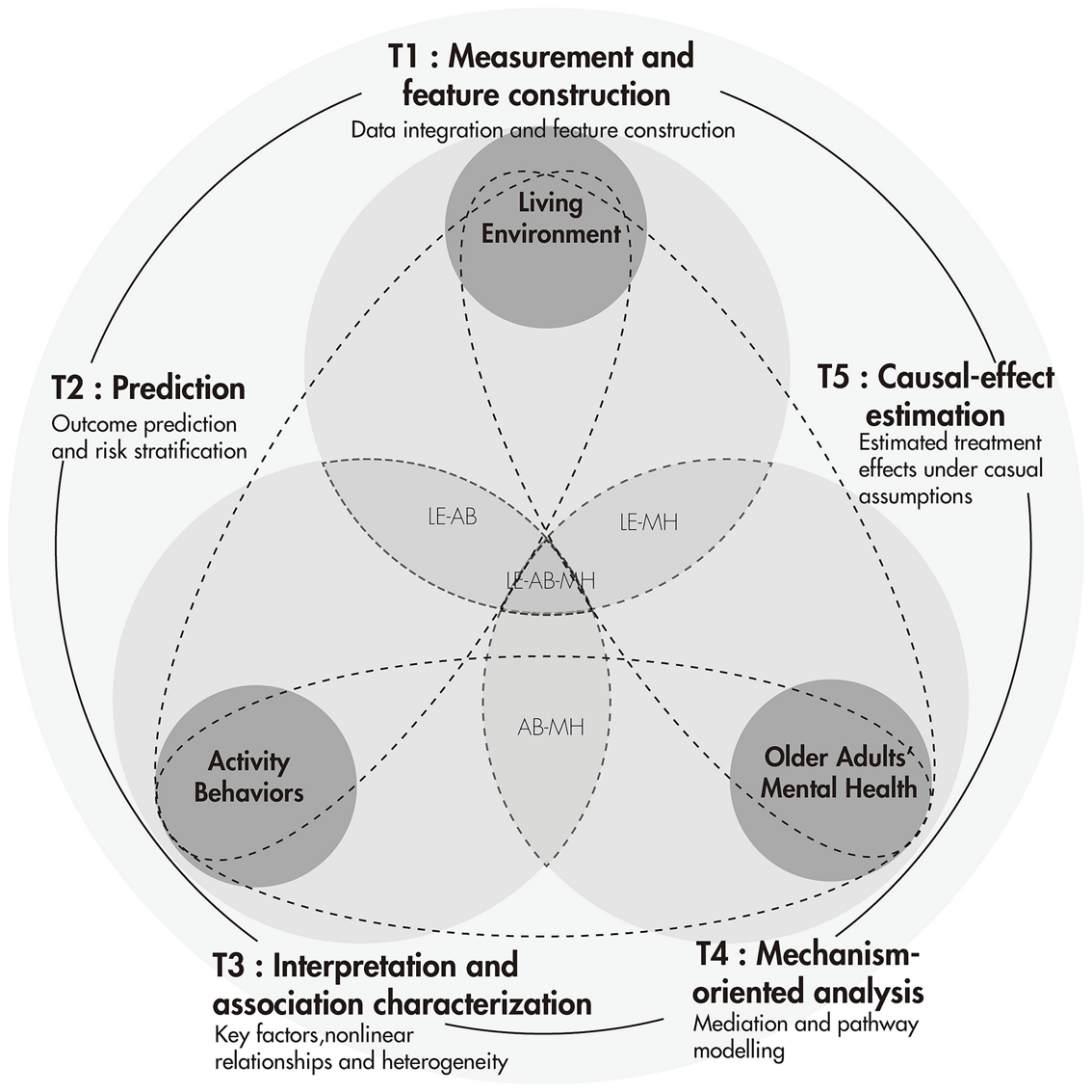
Analytical framework for examining living environment, activity behaviors, and older adults’ mental health.

## 2 Methods

### 2.1 Search strategy

Drawing on the Preferred Reporting Items for Systematic Reviews and Meta-Analyses (PRISMA), this review used a structured process for literature searching, screening, and study inclusion (Figure 2). An initial database search was conducted between September 2024 and May 2026, followed by an updated search on 30 August 2026 using the same databases and search strategies. Eligible records covered studies and reports published from 1 January 2016 to 30 August 2026.

**Figure 2:**
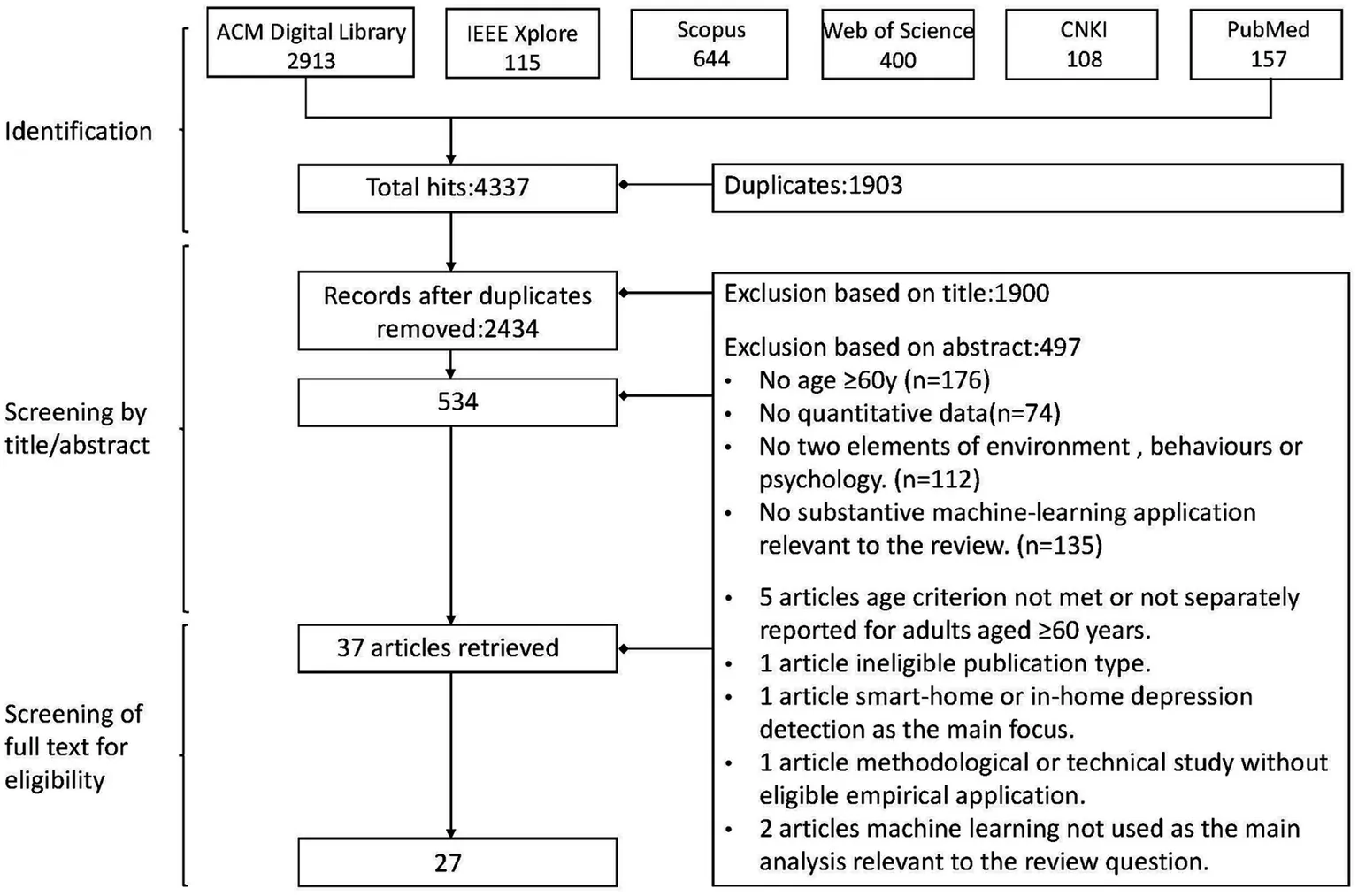
Systematic review search process.

The aim was to synthesize how machine learning has been applied in empirical research on the relationships among living environment, activity behaviors, and mental health in older adults. Searches were conducted in Web of Science Core Collection, Scopus, PubMed, IEEE Xplore, ACM Digital Library, and China National Knowledge Infrastructure (CNKI). Search terms were organized into five concept blocks: older adults, living environment, activity behaviors or social participation, mental health, and machine learning. Within each concept block, synonyms and related terms were combined using OR, whereas different concept blocks were combined using AND. Three relationship-specific searches were conducted to capture studies on living environment-activity behavior, activity behavior-mental health, and living environment-mental health relationships. The full database-specific search strategies, including exact search strings, search fields, Boolean logic, and search dates, are reported in Table 1. No restrictions were imposed on language, country, or geographic setting. Eligible studies were restricted to those published from 1 January 2016 to 30 August 2026.

**Table 1:** Database-specific search strategies and search results.

Database	Search	Search field	Exact search string	Date	Records
Web of Science Core Collection	A: LE–AB	TS	TS = (“older adults” OR “elderly” OR “aging”) AND TS = (“living environment” OR “built environment” OR “residential environment” OR “neighborhood environment”) AND TS = (“physical activity” OR “walking” OR “exercise” OR “mobility” OR “outdoor activity” OR “social participation” OR “social activity”) AND TS = (“machine learning” OR “deep learning” OR “random forest” OR “XGBoost” OR “gradient boosting” OR “support vector machine” OR “neural network” OR “artificial neural network” OR “causal forest” OR “SHAP”)	2026-08-30	116
Web of Science Core Collection	B: AB–MH	TS	TS = (“older adults” OR “elderly” OR “aging”) AND TS = (“physical activity” OR “walking” OR “exercise” OR “mobility” OR “outdoor activity” OR “social participation” OR “social activity”) AND TS = (“mental health” OR “depression” OR “well-being” OR “life satisfaction” OR “psychological well-being”) AND TS = (“machine learning” OR “deep learning” OR “random forest” OR “XGBoost” OR “gradient boosting” OR “support vector machine” OR “neural network” OR “artificial neural network” OR “causal forest” OR “SHAP”)	2026-08-30	246
Web of Science Core Collection	C: LE–MH	TS	TS = (“older adults” OR “elderly” OR “aging”) AND TS = (“living environment” OR “built environment” OR “residential environment” OR “neighborhood environment”) AND TS = (“mental health” OR “depression” OR “well-being” OR “life satisfaction” OR “psychological well-being”) AND TS = (“machine learning” OR “deep learning” OR “random forest” OR “XGBoost” OR “gradient boosting” OR “support vector machine” OR “neural network” OR “artificial neural network” OR “causal forest” OR “SHAP”)	2026-08-30	38
Scopus	A: LE–AB	TITLE-ABS-KEY	TITLE-ABS-KEY(“older adults” OR “elderly” OR “aging”) AND TITLE-ABS-KEY(“living environment” OR “built environment” OR “residential environment” OR “neighborhood environment”) AND TITLE-ABS-KEY(“physical activity” OR “walking” OR “exercise” OR “mobility” OR “outdoor activity” OR “social participation” OR “social activity”) AND TITLE-ABS-KEY(“machine learning” OR “deep learning” OR “random forest” OR “XGBoost” OR “gradient boosting” OR “support vector machine” OR “neural network” OR “artificial neural network” OR “causal forest” OR “SHAP”)	2026-08-30	119
Scopus	B: AB–MH	TITLE-ABS-KEY	TITLE-ABS-KEY(“older adults” OR “elderly” OR “aging”) AND TITLE-ABS-KEY(“physical activity” OR “walking” OR “exercise” OR “mobility” OR “outdoor activity” OR “social participation” OR “social activity”) AND TITLE-ABS-KEY(“mental health” OR “depression” OR “well-being” OR “life satisfaction” OR “psychological well-being”) AND TITLE-ABS-KEY(“machine learning” OR “deep learning” OR “random forest” OR “XGBoost” OR “gradient boosting” OR “support vector machine” OR “neural network” OR “artificial neural network” OR “causal forest” OR “SHAP”)	2026-08-30	465
Scopus	C: LE–MH	TITLE-ABS-KEY	TITLE-ABS-KEY(“older adults” OR “elderly” OR “aging”) AND TITLE-ABS-KEY(“living environment” OR “built environment” OR “residential environment” OR “neighborhood environment”) AND TITLE-ABS-KEY(“mental health” OR “depression” OR “well-being” OR “life satisfaction” OR “psychological well-being”) AND TITLE-ABS-KEY(“machine learning” OR “deep learning” OR “random forest” OR “XGBoost” OR “gradient boosting” OR “support vector machine” OR “neural network” OR “artificial neural network” OR “causal forest” OR “SHAP”)	2026-08-30	60
IEEE Xplore	A: LE–AB	All Metadata	(“older adults” OR “elderly” OR “aging”) AND (“living environment” OR “built environment” OR “residential environment” OR “neighborhood environment”) AND (“physical activity” OR “walking” OR “exercise” OR “mobility” OR “outdoor activity” OR “social participation” OR “social activity”) AND (“machine learning” OR “deep learning” OR “random forest” OR “XGBoost” OR “gradient boosting” OR “support vector machine” OR “neural network” OR “artificial neural network” OR “causal forest” OR “SHAP”)	2026-08-30	13
IEEE Xplore	B: AB–MH	All Metadata	(“older adults” OR “elderly” OR “aging”) AND (“physical activity” OR “walking” OR “exercise” OR “mobility” OR “outdoor activity” OR “social participation” OR “social activity”) AND (“mental health” OR “depression” OR “well-being” OR “life satisfaction” OR “psychological well-being”) AND (“machine learning” OR “deep learning” OR “random forest” OR “XGBoost” OR “gradient boosting” OR “support vector machine” OR “neural network” OR “artificial neural network” OR “causal forest” OR “SHAP”)	2026-08-30	89
IEEE Xplore	C: LE–MH	All Metadata	(“older adults” OR “elderly” OR “aging”) AND (“living environment” OR “built environment” OR “residential environment” OR “neighborhood environment”) AND (“mental health” OR “depression” OR “well-being” OR “life satisfaction” OR “psychological well-being”) AND (“machine learning” OR “deep learning” OR “random forest” OR “XGBoost” OR “gradient boosting” OR “support vector machine” OR “neural network” OR “artificial neural network” OR “causal forest” OR “SHAP”)	2026-08-30	13
ACM DL	A: LE–AB	Anywhere / Metadata	(“older adults” OR “elderly” OR “aging”) AND (“living environment” OR “built environment” OR “residential environment” OR “neighborhood environment”) AND (“physical activity” OR “walking” OR “exercise” OR “mobility” OR “outdoor activity” OR “social participation” OR “social activity”) AND (“machine learning” OR “deep learning” OR “random forest” OR “XGBoost” OR “gradient boosting” OR “support vector machine” OR “neural network” OR “artificial neural network” OR “causal forest” OR “SHAP”)	2026-08-30	526
ACM DL	B: AB–MH	Anywhere / Metadata	(“older adults” OR “elderly” OR “aging”) AND (“physical activity” OR “walking” OR “exercise” OR “mobility” OR “outdoor activity” OR “social participation” OR “social activity”) AND (“mental health” OR “depression” OR “well-being” OR “life satisfaction” OR “psychological well-being”) AND (“machine learning” OR “deep learning” OR “random forest” OR “XGBoost” OR “gradient boosting” OR “support vector machine” OR “neural network” OR “artificial neural network” OR “causal forest” OR “SHAP”)	2026-08-30	1,937
ACM DL	C: LE–MH	Anywhere/Metadata	(“older adults” OR “elderly” OR “aging”) AND (“living environment” OR “built environment” OR “residential environment” OR “neighborhood environment”) AND (“mental health” OR “depression” OR “well-being” OR “life satisfaction” OR “psychological well-being”) AND (“machine learning” OR “deep learning” OR “random forest” OR “XGBoost” OR “gradient boosting” OR “support vector machine” OR “neural network” OR “artificial neural network” OR “causal forest” OR “SHAP”)	2026-08-30	450
CNKI	A: LE–AB	主题	主题 = (老年人 OR 老年 OR 老龄) AND 主题 = (生活环境 OR 建成环境 OR 居住环境 OR 邻里环境) AND 主题 = (身体活动 OR 步行 OR 锻炼 OR 移动 OR 户外活动 OR 社会参与 OR 社会活动) AND 主题 = (机器学习 OR 深度学习 OR 随机森林 OR XGBoost OR 梯度提升 OR 支持向量机 OR 神经网络 OR 人工神经网络 OR 因果森林 OR SHAP)	2026-08-30	39
CNKI	B: AB–MH	主题	主题 = (老年人 OR 老年 OR 老龄) AND 主题 = (身体活动 OR 步行 OR 锻炼 OR 移动 OR 户外活动 OR 社会参与 OR 社会活动) AND 主题 = (心理健康 OR 抑郁 OR 幸福感 OR 生活满意度 OR 心理幸福感) AND 主题 = (机器学习 OR 深度学习 OR 随机森林 OR XGBoost OR 梯度提升 OR 支持向量机 OR 神经网络 OR 人工神经网络 OR 因果森林 OR SHAP)	2026-08-30	51
CNKI	C: LE–MH	主题	主题 = (老年人 OR 老年 OR 老龄) AND 主题 = (生活环境 OR 建成环境 OR 居住环境 OR 邻里环境) AND 主题 = (心理健康 OR 抑郁 OR 幸福感 OR 生活满意度 OR 心理幸福感) AND 主题 = (机器学习 OR 深度学习 OR 随机森林 OR XGBoost OR 梯度提升 OR 支持向量机 OR 神经网络 OR 人工神经网络 OR 因果森林 OR SHAP)	2026-08-30	18
PubMed	A: LE–AB	Title/Abstract	(“older adults”[tiab] OR “elderly”[tiab] OR “aging”[tiab]) AND (“living environment”[tiab] OR “built environment”[tiab] OR “residential environment”[tiab] OR “neighborhood environment”[tiab]) AND (“physical activity”[tiab] OR “walking”[tiab] OR “exercise”[tiab] OR “mobility”[tiab] OR “outdoor activity”[tiab] OR “social participation”[tiab] OR “social activity”[tiab]) AND (“machine learning”[tiab] OR “deep learning”[tiab] OR “random forest”[tiab] OR “XGBoost”[tiab] OR “gradient boosting”[tiab] OR “support vector machine”[tiab] OR “neural network”[tiab] OR “artificial neural network”[tiab] OR “causal forest”[tiab] OR “SHAP”[tiab])	2026-08-30	18
PubMed	B: AB–MH	Title/Abstract	(“older adults”[tiab] OR “elderly”[tiab] OR “aging”[tiab]) AND (“physical activity”[tiab] OR “walking”[tiab] OR “exercise”[tiab] OR “mobility”[tiab] OR “outdoor activity”[tiab] OR “social participation”[tiab] OR “social activity”[tiab]) AND (“mental health”[tiab] OR “depression”[tiab] OR “well-being”[tiab] OR “life satisfaction”[tiab] OR “psychological well-being”[tiab]) AND (“machine learning”[tiab] OR “deep learning”[tiab] OR “random forest”[tiab] OR “XGBoost”[tiab] OR “gradient boosting”[tiab] OR “support vector machine”[tiab] OR “neural network”[tiab] OR “artificial neural network”[tiab] OR “causal forest”[tiab] OR “SHAP”[tiab])	2026-08-30	131
PubMed	C: LE–MH	Title/Abstract	(“older adults”[tiab] OR “elderly”[tiab] OR “aging”[tiab]) AND (“living environment”[tiab] OR “built environment”[tiab] OR “residential environment”[tiab] OR “neighborhood environment”[tiab]) AND (“mental health”[tiab] OR “depression”[tiab] OR “well-being”[tiab] OR “life satisfaction”[tiab] OR “psychological well-being”[tiab]) AND (“machine learning”[tiab] OR “deep learning”[tiab] OR “random forest”[tiab] OR “XGBoost”[tiab] OR “gradient boosting”[tiab] OR “support vector machine”[tiab] OR “neural network”[tiab] OR “artificial neural network”[tiab] OR “causal forest”[tiab] OR “SHAP”[tiab])	2026-08-30	8

### 2.2 Eligibility criteria

The initial search identified 4,337 records, of which 2,434 remained after duplicate removal. A total of 1900 weakly relevant records were excluded after title and abstract screening, leaving 534 records for abstract screening. Only empirical studies reporting original data and a substantive analytical application of machine learning were eligible. Reviews, editorials, commentaries, protocols, conference abstracts, and purely methodological studies without an empirical application were excluded. Abstracts were assessed against four criteria. (1) included participants aged 60 years or older, or reported separately extractable findings for this age group; (2) empirically examined relationships involving at least two of the three domains of living environment, activity behaviors, and mental health; (3) included at least one machine-learning approach; and (4) used machine learning as a substantive component of the empirical analytical workflow, rather than mentioning it only conceptually or using it solely as a peripheral technical procedure.

Full-text assessment was conducted according to four exclusion principles. Studies were excluded if they did not meet the age criterion or did not report separately extractable findings for adults aged 60 years or older (46–50). Studies were also excluded if they were ineligible publication types, such as conference abstracts, protocols, editorials, commentaries, or reviews (51). Further exclusions were made when machine learning was not used as the main analytical method relevant to the review question (52, 53), when the article was primarily a methodological or technical study without an eligible empirical application (54), or when the study focused mainly on smart-home or in-home depression detection rather than relationships involving living environment, activity behaviors, or mental health (55). A final set of 27 studies met the inclusion criteria.

### 2.3 Operational definition of activity behaviors

Activity behaviors were defined as observable or self-reported activities through which older adults act within, move through, or interact with their living environments. For coding purposes, activity behaviors were classified into four subdomains: physical activity behaviors AB(P), mobility behaviors AB(M), social activity behaviors AB(S), and public-space activity/use behaviors AB(U).

AB(P) included walking, exercise, physical activity, and active travel; AB(M) included driving, travel behavior, and daily mobility; AB(S) included social participation, community or group activities, volunteering involving interpersonal contact, and recreational activities undertaken with others; and AB(U) included pedestrian activity and the actual use of parks, streets, and other public spaces.

Social support, social cohesion, loneliness, functional capacity, activities of daily living were not coded as activity behaviors unless an explicit behavioral component was separately measured.

### 2.4 Study selection, data extraction, and synthesis

Title and abstract screening was conducted independently by two reviewers according to the predefined eligibility criteria. Full-text screening was subsequently conducted independently by the same two reviewers. Disagreements between the two reviewers at either stage were resolved through discussion until consensus was reached.

Data extraction was performed by one reviewer using a standardized extraction form and was subsequently checked by the second reviewer for completeness and consistency. The extracted information included bibliographic details, study area, sample characteristics, data sources, operational definitions of living environment, activity behaviors, and mental health, machine-learning methods, validation approaches, relationship types, and the role of machine learning in the analytical workflow.

The functional categories of machine-learning roles were developed iteratively during an initial reading of the eligible studies and pilot data extraction. Based on the analytical purposes observed across the studies, four categories were established: measurement, prediction, interpretation, and causal-inference support. These categories were finalized before the formal evidence synthesis and then applied consistently during data extraction and coding. Multi-label coding was used when a study employed machine learning for more than one analytical function. When a single primary function was required, it was determined according to the study’s stated objective and the function most directly linked to its main outcome or inference. If no single dominant function could be identified, the study was retained as multi-label rather than being assigned an arbitrary primary category. All task classifications were checked by the second reviewer, and disagreements were resolved through discussion until consensus was reached.

Based on the functional roles observed during the initial reading and pilot extraction of the eligible studies, five task categories were established: (T1) measurement and feature construction, including the extraction or construction of environmental, behavioral, and other analytical variables from raw or complex data; (T2) prediction of behavioral, mental-health, and other study outcomes; (T3) interpretation and association characterization, including the assessment of variable importance, nonlinear associations, thresholds, interactions, and heterogeneous patterns; (T4) pathway and mechanism-oriented analysis, involving the examination of potential pathways or mechanisms linking study variables; and (T5) causal-effect estimation, involving the estimation of average or heterogeneous treatment effects using explicit causal estimators or designs under stated causal assumptions. Studies using mediation analysis, structural equation modeling, or pathway modeling were classified as T4 unless they explicitly applied causal estimators designed for treatment-effect estimation under causal assumptions. These categories were not mutually exclusive. Studies in which machine learning served multiple analytical functions were therefore assigned multiple task labels (Table 2).

**Table 2:** Articles included in the systematic review.

Author(s) and year	Study area	Sample characteristics	Core data types and sources	Relationship type	Task definition	Main ML method(s)	Methodological role of ML	Standardized ML task code(s)	Validation and analytical approach
Liu and Liao (5)	Chongqing, China	N = 1,185 respondents aged over 60 from 43 communities	WHO-5 Well-Being Index; survey; 500-m buffers; blue space; parks; NDVI; street-view greenness; perceived greenery; facility quality/quantity; neighborhood disorder; housing data	LE-MH	Modeled older adults’ mental well-being and ranked natural/built-environment predictors while considering housing self-selection	XGBoost; SHAP	Outcome modeling plus association characterization and model interpretation	T2; T3	XGBoost modeling with SHAP; public/private housing stratification; VIF check; cross-sectional; no external validation
Helbich et al. (6)	Beijing, China	N = 1,190 participants aged 60 or above	Questionnaire data; GDS-15; Tencent Street View; FCN-derived street-view green/blue space; NDVI; NDWI; GlobeLand30 land cover	LE-MH	Compared street-view and satellite-derived green/blue-space exposure metrics and examined associations with geriatric depression	Fully convolutional neural network; multilevel regression	Measurement/feature construction; association characterization	T1; T3	Street-view exposure extraction; multilevel regression with robustness tests
Bi and Chen (8)	China	N = 2,086 older adults after excluding respondents under 60 years from CHARLS-linked data	CHARLS; air-pollution exposure data; depression symptoms; demographic, health, social and behavioral covariates	LE-MH	Predicted depression risk and examined pathways linking air pollution and individual factors to depression among older adults	Logistic regression; random forest; SVM; GBDT; XGBoost; GBM; LightGBM; DNN; RNN; LSTM; SHAP; SEM	Outcome prediction, association interpretation and pathway/mechanism-oriented analysis	T2; T3; T4	Trained ten ML models; used SHAP for variable importance; used SEM to examine mediating pathways
Asadollahi et al. (60)	Shiraz, Iran	N = 3,000 older persons aged 60+	Municipal environmental data; green space per capita; population density; waste production; BMI; frailty; depression; anxiety; life satisfaction	LE-MH	Examined urban environmental impacts on older adults’ health and developed predictive ML models for health outcomes	Decision Tree; SVM; regression models	Outcome prediction plus association characterization	T2; T3	Cross-sectional modeling; SVM reported better predictive performance than decision trees
Yue et al. (7)	Dalian, China	N = 879 respondents aged 60 years or older	Survey data; mental well-being measures; NDVI; vegetation coverage; park coverage; street-view greenspace; environmental perceptions	LE-MH	Examined associations between multiple greenspace measures and mental health among Chinese older adults, including mediating effects of environmental perceptions	Machine-learning-based street-view greenspace extraction; multilevel regression	Measurement/feature construction and pathway- or mechanism-oriented analysis	T1; T4	Derived eye-level greenspace measures from street-view imagery using ML and examined mediation through environmental perceptions
Li et al. (61)	Jintang County, China	Rural older adults	Questionnaire data; self-reported average daily walking duration and other daily activity-duration measures; subjective built-environment measures; well-being data	LE-AB(P)-MH	Explored non-linear effects of walking accessibility on subjective well-being in rural older adults	Random Forest	Association characterization and model interpretation	T3	Random-forest variable importance and nonlinear-effect interpretation
Zhao et al. (3)	China	N = 4,627 individuals aged 60 years and above from CHARLS 2020 merged with the 2014 Life History Survey	CHARLS; life satisfaction; depressive symptoms; social activity participation; housing/material conditions; life-course and psychosocial variables	LE-AB(S)-MH	Examined determinants of life satisfaction among older adults using integrated medical statistics and machine learning	Generalized ordered logit regression; XGBoost	Outcome prediction and nonlinear association characterization	T2; T3	Combined GOLR with XGBoost to capture linear and nonlinear associations and conduct individual-level prediction
Wang and Yao (62)	Shanghai, China	Older adults aged over 65 from WHO SAGE Shanghai sample	Street-view images; visual green-space quantity/quality; depression measures; perceived stress; physical activity; PM2.5	LE-AB(P)-MH	Examined pathways linking visual green space to depression via stress, physical activity, social cohesion and air pollution	Machine-learning street-view green-space extraction; regression/mediation models	Measurement/feature construction; pathway- or mechanism-oriented analysis	T1; T4	Multilevel logistic/linear regression and mediation/pathway analysis; ML used to calculate street-view green-space exposure
Su et al. (16)	Xiamen, China	N = 400 community-dwelling older-adult volunteers aged 60 years or older; face-to-face safety-perception survey conducted from 20 February to 2 March 2025	Multi-scale built environment; cumulative opportunity measures; street-scene semantic segmentation; XGBoost; GeoShapley; Baidu API/service accessibility	LE-AB(P/M)	Assessed multi-scale environmental configurations for older adults’ walkability with explainable machine learning	Street-scene semantic segmentation; XGBoost; GeoShapley	Measurement/feature construction; association characterization and model interpretation	T1; T3	CNN-BiLSTM used an 80:20 training/evaluation split (R2 = 0.867; MAE = 0.864). XGBoost hyperparameters were optimized by cross-validation and evaluated using R2, MAE, and RMSE (reported ranges: R2 = 0.806–0.906, MAE = 0.856–0.910, RMSE = 0.97–1.14); no independent external validation reported
Wang et al. (18)	Zhongshan, China	N = 4,784 adults aged 60 years or older from 274 urban and rural neighborhoods in the 2012 Zhongshan Household Travel Survey	Built-environment indicators; cycling-frequency data	LE-AB(P)	Examined nonlinear associations between the built environment and cycling frequency	XGBoost; gradient boosting decision tree; multiple linear regression; partial dependence plots	Association characterization and model interpretation	T3	Compared XGBoost with gradient boosting decision tree and multiple linear regression using MSE, MAE, and MAPE; XGBoost produced the lowest reported errors; default XGBoost parameters were used; no independent external validation reported
Cheng et al. (19)	Nanjing, China	N = 702 adults aged 60 years or older from the 2013 Nanjing Household Travel Survey (376 men and 326 women)	Built-environment indicators; older adults’ walking data	LE-AB(P)	Examined non-linear built-environment effects on older adults’ walking	Random Forest	Association characterization and model interpretation	T3	Random forest tuned to 500 trees, five splitting variables, and maximum tree depth 1,200; OOB performance was RMSE = 32.20 and pseudo-R2 = 0.313. Ten-fold cross-validation compared random forest with linear regression using out-of-sample MAE and RMSE (random forest: MAE = 21.23, RMSE = 32.15; linear regression: MAE = 27.45, RMSE = 39.64); no independent external validation reported
Wu et al. (20)	Zhongshan City, China	N = 4,329 adults aged 60 years or older from the stratified 2012 Zhongshan Household Travel Survey (2,979 aged 60–69 years and 1,350 aged 70 years or older)	Built-environment indicators; walking-duration data; age and retirement variables	LE-AB(P)	Examined nonlinear and threshold effects of built environment on walking duration	Gradient boosting decision trees (GBDT; R gbm package); partial dependence plots	Association characterization and model interpretation	T3	Five-fold cross-validation tuned tree depth, learning rate, and number of trees by minimizing RMSE; the final GBDT used tree depth 45, learning rate 0.001, and 3,670 trees. Age- and retirement-status subgroup analyses were performed; no independent external validation reported
Zang et al. (21)	Lanzhou, China	N = 1,773 adults aged 60 years or older with valid 2021 medical-examination records from 14 areas in Chengguan and Qilihe districts, Lanzhou	Built-environment indicators; light physical-activity data	LE-AB(P)	Examined nonlinear effects of the built environment on light physical activity	Random forest regression (scikit-learn; GridSearchCV); linear regression comparison; partial dependence plots	Association characterization and model interpretation	T3	GridSearchCV optimized the random-forest parameters; ten-fold cross-validation compared random forest with linear regression using MAE and RMSE, followed by Wilcoxon tests. Random forest had lower median errors (MAE = 0.486 versus 0.492; RMSE = 0.492 versus 0.496; both *p* = 0.005); no independent external validation reported
Zhu et al. (23)	Shanghai, China	Older adults	Neighborhood environment; personal perception; walking-behavior data	LE-AB(P)	Examined interactions between neighborhood environment and personal perception affecting walking behavior	Random Forest; human-machine adversarial framework	Association characterization and model interpretation	T3	RF interaction analysis with human-machine adversarial framework
Wang et al. (4)	Zhongshan, China	N = 4,329 adults aged 60–95 years from the stratified 2012 Zhongshan Household Travel Survey (2,616 men and 1,713 women)	Built environment; social environment; bus-use behavior	LE-AB(M)	Examined non-linear effects of built and social environment on bus use among older adults	XGBoost	Association characterization and model interpretation	T3	Compared XGBoost with multiple linear regression using testing pseudo-R2 (0.838 for XGBoost versus 0.160 for multiple linear regression); partial dependence plots were used to characterize nonlinear and threshold effects; no independent external validation reported
Hafezifar et al. (17)	St. Louis City/County, USA	Older drivers aged 65+; n = 134	GIS built-environment measures; transit accessibility; land-use mix; road network; driving distance/trip frequency/short-long trip ratio	LE-AB(M)	Examined how neighborhood built environment affects driving behaviors of older adults	Gradient Boosting Regressor (scikit-learn); decision tree, random forest, and SVM regression comparisons; feature importance and partial dependence plots	Feature construction and association characterization	T1; T3	Data were split 70:30 into training and test sets; three-fold cross-validation within the training data and grid search tuned tree count, learning rate, and maximum depth by minimizing RMSE. GBM was selected for interpretability (total-distance RMSE = 2,196.92; SVM regression had the lowest comparison RMSE of 2,084.84); no independent external validation reported
Torku et al. (66)	Hung Hom, Hong Kong, China	N = 10 ambulatory adults aged 65 years or older; model results reported usable physiological records for 9 participants	Multimodal physiological sensing; neighborhood mobility data; risk-hotspot information	LE-AB(M)-MH	Detected stressful older adult-environment interactions to improve neighborhood mobility	Decision tree; Gaussian SVM; k-nearest neighbour; ensemble bagged tree; deep belief network; multimodal sensor fusion; spatial relative-risk hotspot analysis	Feature construction; outcome/event detection; model interpretation	T1; T2; T3	Random under-sampling was repeated 20 times to create 20 balanced train/test splits; ten-fold cross-validation evaluated accuracy, precision, and recall. Ensemble bagged tree performed best (98.13% accuracy for stress/non-stress and 98.25% for low/high stress); hotspot power analysis used 10,000 simulations with a 0.80 threshold; no independent external validation reported
Kim et al. (31)	Incheon, South Korea	N = 47 community-dwelling older adults living alone, aged 65–94 years, analyzed from 56 recruited participants	Ecological momentary assessment; Actiwatch data; depression-related measures	AB(P)-MH	Predicted depression among older adults living alone using EMA and wearable activity data	Binary logistic regression; machine-learning logit model; decision tree; boosted trees; random forest	Feature construction from EMA/wearable data; depression prediction	T1; T2	Random 65:35 training/test split (n = 30/n = 17) with class balance maintained; bootstrapped data partitioning and model training repeated 100 times; evaluated using accuracy, precision, recall/sensitivity, specificity, F score, and AUC; no independent external validation
Zhang et al. (58)	China	N = 16,627 participants aged 60 years or above from CHARLS wave 4	CHARLS; depressive symptoms; chronic diseases; cognition; ADL; social support; physical exercise-related variables	AB(P)-MH	Explored influential factors and pathways of depression among older adults and examined heterogeneity across age groups	PLS-SEM; random forest; XGBoost; causal forest	Association characterization, pathway analysis and causal-effect-oriented heterogeneity assessment	T3; T4; T5	Used PLS-SEM and random forest modeling; applied XGBoost for age-stratified importance maps and causal forest for intervention-oriented heterogeneity
Wang and Pan (63)	Rural China	N = 3,232 rural older adults from CHARLS 2018	CHARLS; CES-D10 depressive symptoms; social, psychological and physiological factors; exercise and social activity-related variables	AB(P)-MH	Developed and validated ML models for predicting depression risk among rural older adults	LASSO; SVM; DMR-CNN; decision tree; XGBoost; random forest; logistic regression; SHAP	Outcome prediction/classification and model interpretation	T2; T3	Used LASSO for predictor selection; compared six ML algorithms with ROC, calibration and decision curve analysis; interpreted the best model with SHAP
Lin et al. (32)	Xiamen, China	N = 50 adults aged 63–81 years selected from 92 recruited participants and 84 valid questionnaire respondents (25 with and 25 without depressive symptoms; 13 men and 37 women)	Spatiotemporal gait features; depression-recognition data	AB(P)-MH	Recognized depression among older adults using spatiotemporal gait features	Logistic regression; gradient boosting decision tree; support vector machine; random forest; SHAP	Feature construction; outcome recognition/classification; interpretation	T1; T2; T3	Random 70:30 training/test split; ten-fold cross-validation and hyperparameter tuning were performed within the training data, and testing was repeated 1,000 times. Evaluation used sensitivity, PPV, accuracy, AUC-ROC, Brier score, and decision curve analysis; random forest performed best (AUC-ROC = 0.911; accuracy = 0.867); no independent external validation reported
Wang et al. (64)	China	N = 5,267 Chinese older adults from CHARLS 2018	CHARLS; depressive symptoms; physical activity; social activity; demographic, socioeconomic and health variables	AB(P)-MH	Examined urban–rural differences in key factors associated with depressive symptoms among Chinese older adults	Random forest; logistic regression	Association characterization and variable-importance interpretation	T3	Used random forest to identify key predictors and logistic regression to estimate associations in urban and rural subgroups
Tan et al. (59)	China	N = 10,502 Chinese older adults aged 60 years or older from CLASS	China Longitudinal Aging Social Survey (CLASS); depressive symptoms; health status; psychosocial needs; ADL and social involvement/exclusion indicators	AB(S)-MH	Developed an ML-based screening model for depression risk and identified health-related, psychosocial and ADL predictors among older adults	Bagged tree; RDA; logistic regression; MARS; ANN; random forest; SVM; XGBoost	Outcome prediction/classification and feature relevance interpretation	T2; T3	Compared eight supervised ML models after preprocessing; used information-gain-based feature selection and model performance metrics
Takemura et al. (29)	Japan	JAGES cohort; propensity-score-matched older adults aged 65+; n = 11,146	JAGES 2016/2019/2022 waves; direct social participation; GDS depressive symptoms; sociodemographic/behavioral/health variables	AB(S)-MH	Estimated heterogeneity in the association/effect of social participation on depressive symptoms	Propensity score matching; causal forest	Causal-effect estimation and heterogeneity analysis	T5; T3	1:1 propensity score matching; causal forest; CATE estimated; high-benefit/low-benefit groups compared
Song et al. (65)	China	Older adults aged 60 years or older from CHARLS 2020	CHARLS; depression; demographic and health variables; behavioral factors including smoking, drinking, sleep, social activities and weekly physical exercise	AB(P/S)-MH	Predicted depression among older adults and interpreted key predictors using machine-learning algorithms	Logistic regression; KNN; SVM; decision tree; LightGBM; random forest; SHAP	Outcome prediction/classification and model interpretation	T2; T3	Compared multiple ML models with ROC/Delong testing and decision curve analysis; interpreted model outputs with SHAP
Site et al. (30)	Tampere region, Finland	Six female older participants aged 68–87; sensor data for five participants	AISOLA surveys; LSNS-6; UCLA 3-item Loneliness Scale; Pozyx UWB indoor positioning; GPS; accelerometer-derived mobility features	AB(M)-MH	Classified loneliness/social-isolation levels from indoor, outdoor and combined mobility patterns; explored links between mobility features and loneliness metrics	SVM; Random Forest; XGBoost	Feature construction from mobility sensors; outcome classification/prediction; feature relevance interpretation	T1; T2; T3	Compared indoor/outdoor/combined models with accuracy, confusion matrices, RMSE, MAE and R2; no external validation reported
Jiang et al. (22)	Suzhou, China	N = 1,020 older adults	Survey; weekly physical and social/recreational activity duration/frequency; 11 built-environment indicators; OSM; Baidu Street View; GluonCV-derived greening/sky rates; POI/accessibility; WorldPop	LE-AB(P/S)	Examined nonlinear and threshold effects of built environment on physical and social/recreational activity durations	XGBoost; GluonCV image parsing; linear regression baseline; PDP	Measurement/feature construction; association characterization and model interpretation	T1; T3	Grid search with five-fold cross-validation; RMSE and pseudo-R2; XGBoost compared with linear regression

Following study selection, data were extracted using a standardized coding table. The extracted information included author(s) and year, study area, sample characteristics, core data types and sources, relationship type, task description, main machine-learning method(s), the methodological role of machine learning, and the validation and analytical approach.

Given the substantial heterogeneity in study designs, exposure and outcome measurements, machine-learning functions, and reported effect measures, quantitative meta-analysis was not considered appropriate. The evidence was therefore synthesized narratively using a structured cross-study comparison.

### 2.5 Risk-of-bias and applicability assessment

Risk-of-bias assessment was conducted only after the eligibility re-audit. Studies that did not meet the target-population, empirical-sample, publication-type, or relationship-domain inclusion criteria were excluded before appraisal rather than retained and downgraded because of applicability concerns.

Design-specific JBI (56) critical appraisal tools were applied to analytical cross-sectional and cohort studies. PROBAST+AI (57) was applied to eligible prediction-model studies, with methodological quality and applicability assessed for model-development components and risk of bias and applicability assessed for model-evaluation components. One reviewer completed the initial assessments, and the second reviewer systematically checked all domain-level ratings, overall judgements, and supporting rationales against the full texts. Disagreements were resolved through discussion until consensus was reached (56, 57).

Domain-level results and the decision rules used to derive overall judgements are reported in Table 3. For PROBAST+AI, an overall high-risk judgement was assigned when at least one domain was rated high risk, whereas a some-concerns judgement was assigned when at least one domain raised concerns and no domain was rated high risk. Review-specific decision rules for the JBI assessments, including the domains treated as critical, were defined before the final reassessment and are presented in Table 3.

**Table 3:** Risk-of-bias and applicability assessment of included studies.

Study	Design	Sample	Age range	Appraisal tool	Eligibility status	Overall risk/quality judgement	Applicability concern	Key basis for judgement	Second-author verification
Liu and Liao (5)	Cross-sectional study	1,185 respondents from 43 communities	Older than 60 years	JBI cross-sectional	Included	Some concerns	Low concern	Reasonable sample and confounder adjustment, but cross-sectional design, mixed subjective/objective exposures, and residual housing self-selection remain.	Confirmed
Helbich et al. (6)	Cross-sectional population-based study	1,190 participants	60 years or older	JBI cross-sectional	Included	Some concerns	Low concern	Population-based sampling, validated GDS-15, and sensitivity analyses are strengths; cross-sectional exposure and residential self-selection remain.	Confirmed
Bi and Chen (8)	Longitudinal secondary analysis with SEM	2,086 older adults from CHARLS-linked data	60 years or older	JBI cohort/cross-sectional	Included	Some concerns	Low concern	Integrates environmental exposure, depression prediction, SHAP and SEM, but causal pathway wording should remain cautious.	Confirmed
Asadollahi et al. (60)	Cross-sectional stratified survey	3,000 participants across 11 municipal zones	60 years or older	PROBAST+AI	Included	High risk	Low concern	Large stratified sample and validated outcomes, but only internal geographic validation and limited calibration/transportability evidence for prediction models.	Confirmed
Yue et al. (7)	Cross-sectional survey	879 respondents in Dalian	60 years or older	JBI cross-sectional	Included	Some concerns	Low concern	Multiple greenspace measures and mental-health outcomes are reported, but cross-sectional mediation and local sampling limit causal interpretation.	Confirmed
Li et al. (61)	Cross-sectional survey	515 respondents; 30 incomplete questionnaires excluded	60 years or older	JBI cross-sectional	Included	Some concerns	Low concern	Local rural sample and cross-sectional outcome; potential residual confounding despite demographic adjustment.	Confirmed
Zhao et al. (3)	Cross-sectional/life-course secondary analysis	4,627 CHARLS participants	60 years or older	JBI cross-sectional	Included	Some concerns	Low concern	National survey data and XGBoost interpretation are appropriate, but life-course recall and cross-sectional outcome assessment leave residual confounding.	Confirmed
Wang and Yao (62)	Cross-sectional secondary analysis	WHO SAGE Shanghai older-adult sample	Older than 65 years	JBI cross-sectional	Included	High risk	Low concern	Depression measured with two self-reported questions; cross-sectional mediation and residential self-selection limit causal interpretation.	Confirmed
Su et al. (16)	Cross-sectional volunteer survey	400 volunteers	60 years or older	JBI cross-sectional	Included	High risk	Some concerns	Local volunteer sampling and subjective safety-perception outcome; nonresponse, confounding, and missing-data handling are insufficiently reported.	Confirmed
Wang et al. (18)	Cross-sectional travel survey	4,784 respondents from 274 neighborhoods	60 years or older	JBI cross-sectional	Included	Some concerns	Low concern	Large stratified sample; cycling was self-reported for one day and unmeasured confounding remains.	Confirmed
Cheng et al. (19)	Cross-sectional travel survey	702 respondents from 5,172 complete survey responses	60 years or older	JBI cross-sectional	Included	Some concerns	Low concern	Built-environment measures and adjustment are reasonable; walking is one-day self-report and recruitment/nonresponse may introduce selection bias.	Confirmed
Wu et al. (20)	Cross-sectional travel survey	4,329 respondents; stratified random sample	60 years or older	JBI cross-sectional	Included	Some concerns	Low concern	Large stratified sample and cross-validation; self-reported walking and cross-sectional residual confounding remain.	Confirmed
Zang et al. (21)	Cross-sectional survey	1,773 respondents	60 years or older	JBI cross-sectional	Included	Some concerns	Low concern	Reasonable sample and ten-fold validation; self-reported activity and incomplete control of contextual confounding remain.	Confirmed
Zhu et al. (23)	Cross-sectional ecological/aggregated study	169,996 older residents aggregated to 995 neighborhoods	60 years or older	JBI cross-sectional	Included	High risk	Some concerns	Neighborhood-level aggregation of individual behavior creates ecological-bias risk; data provenance, missingness, and confounder control need clearer reporting.	Confirmed
Wang et al. (4)	Cross-sectional travel survey	4,329 respondents; stratified random sample	60–95 years	JBI cross-sectional	Included	Some concerns	Low concern	Large probability-based sample and appropriate modeling; one-day self-reported travel and residual confounding remain.	Confirmed
Hafezifar et al. (17)	Naturalistic driving observational study	134 cognitively normal active drivers	65 years or older	JBI cross-sectional	Included	Some concerns	High concern	Objective GPS outcomes are a strength; selected cognitively normal frequent drivers and minimum-trip requirements limit representativeness.	Confirmed
Torku et al. (66)	Multimodal physiological field study	10 recruited; analytic physiological data from 9	65 years or older	PROBAST+AI	Included	High risk	High concern	Extremely small participant sample; 5,518 repeated observations may inflate effective sample size and observation-level cross-validation may leak participant information.	Confirmed
Kim et al. (31)	Prospective observational sensor study	47 analyzed (56 enrolled); 94% women	60 years or older; mean 78	PROBAST+AI	Included	High risk	High concern	Convenience sample, small test set (n = 17), strong sex imbalance, no external validation.	Confirmed
Zhang et al. (58)	Cross-sectional secondary analysis	16,627 CHARLS participants	60 years or older	JBI cross-sectional	Included	Some concerns	Low concern	Large older-adult sample and multiple analytical approaches, but cross-sectional design and self-reported variables limit causal interpretation.	Confirmed
Wang and Pan (63)	Cross-sectional prediction-model study	3,232 rural older adults from CHARLS	60 years or older	PROBAST+AI	Included	High risk	Low concern	Predictive workflow is explicit and interpreted with SHAP, but validation is internal and based on secondary survey data.	Confirmed
Lin et al. (32)	Cross-sectional study with outcome-stratified subsampling	50 analyzed (25 depression, 25 non-depression) from 92 recruited	Older than 60 years	PROBAST+AI	Included	High risk	High concern	Outcome-balanced selected subsample, small train/test split, and no independent external validation.	Confirmed
Wang et al. (64)	Cross-sectional secondary analysis	5,267 CHARLS participants	60 years or older	JBI cross-sectional	Included	Some concerns	Low concern	Random forest identified key predictors across urban/rural subgroups, but causal interpretation is limited by cross-sectional design and residual confounding.	Confirmed
Tan et al. (59)	Cross-sectional secondary analysis	10,502 older adults from CLASS	60 years or older	PROBAST+AI	Included	High risk	Low concern	Large sample and clear depression outcome, but model development relies on internal validation and secondary survey predictors.	Confirmed
Takemura et al. (29)	Longitudinal cohort study	11,000 + matched/analytic older adults in JAGES	65 years or older	JBI cohort	Included	Some concerns	Low concern	Longitudinal exposure/outcome ordering, PSM, AIPW and sensitivity analyses are strengths; 45.1% attrition and residual unmeasured confounding remain.	Confirmed
Song et al. (65)	Cross-sectional prediction-model study	CHARLS 2020 older-adult sample	60 years or older	PROBAST+AI	Included	High risk	Low concern	Multiple ML models and SHAP interpretation were used, but prediction performance was internally evaluated without external validation.	Confirmed
Site et al. (30)	Sensor-based feasibility study	6 women; sensor data available for 5	68–87 years	PROBAST+AI	Included	High risk	High concern	Very small convenience sample; repeated sensor observations may not be independent; no participant-level external validation.	Confirmed
Jiang et al. (22)	Cross-sectional survey	1,020 valid responses from 1,070 recruited	65 years or older	JBI cross-sectional	Included	Some concerns	Some concerns	Stratified recruitment and validation are reported, but excluding severe cognitive or mobility difficulty narrows representativeness.	Confirmed

## 3 Results

### 3.1 Study selection and basic characteristics

A total of 27 studies met the eligibility criteria and were included in the synthesis (3–8, 16–23, 29–32, 58–66). The studies were published between 2019 and 2026, with 20 studies (74.1%) published from 2024 onwards (3–8, 16–23, 29–32, 58–66). Geographically, 22 studies (81.5%) were conducted in China, while one study each (3.7%) was conducted in Iran, the United States, South Korea, Japan, and Finland (3–8, 16–23, 29–32, 58–66). Cross-sectional designs predominated, accounting for 21 studies (77.8%), whereas six studies (22.2%) used longitudinal, cohort, observational, case–control-like, or feasibility-based designs (3–8, 16–23, 29–32, 58–66).

Regarding the relationships examined, nine studies (33.3%) focused on LE–AB relationships (4, 16, 18–23, 62), nine (33.3%) on AB–MH relationships (17, 29, 31, 32, 58, 59, 63, 64, 66), and five (18.5%) on LE–MH relationships (5–8, 60). Four studies (14.8%) simultaneously examined relationships involving all three domains, LE–AB–MH (3, 7, 17, 61). Because some studies addressed more than one domain or relationship, these categories should be interpreted according to the primary relationship coding shown in Table 2.

Sample sizes varied substantially across the included studies, ranging from small sensor- or feasibility-based samples of fewer than 10 participants to large secondary or aggregated datasets involving thousands of older adults. Most studies examined community-dwelling adults aged 60 years or older, while several focused on specific subgroups, including rural residents, cognitively normal older drivers, older adults living alone, and participants with or without depressive symptoms. The included evidence therefore covered both general older-adult populations and selected subgroups, although the latter may have limited representativeness for the broader older population.

The included studies varied substantially in sample size, including several small sensor-based feasibility studies and large survey-based analyses involving more than 16,000 older adults. One ecological study aggregated data from 169,996 older residents across 995 neighborhoods. Most studies used cross-sectional survey or secondary-data designs, whereas a smaller number used longitudinal cohorts, naturalistic driving observations, physiological or wearable sensors, and feasibility-based designs.

The included studies examined different parts of the living environment–activity behavior–mental health framework. Most focused on pairwise LE–AB, AB–MH, or LE–MH relationships, while a smaller group incorporated all three domains. Within the activity-behavior domain, physical activity behaviors [AB(P)]—particularly walking and physical activity—were most frequently represented (16, 18–23, 62), followed by mobility behaviors [AB(M)] such as travel, driving, and movement patterns (4, 17, 30, 66); social/recreational activity behaviors [AB(S)] appeared less frequently (22, 29). The evidence was distributed across partially overlapping relationships among LE, AB, and MH, with pairwise relationships more commonly examined than the three domains simultaneously.

### 3.2 Machine-learning task categories and commonly used models

Across the 27 included studies, 10 studies (37.0%) were classified under measurement and feature construction (T1), 11 (40.7%) under prediction (T2), 24 (88.9%) under interpretation and association characterization (T3), four (14.8%) under pathway and mechanism-oriented analysis (T4), and two (7.4%) under causal-effect estimation (T5). Because multi-label coding was used, the categories were not mutually exclusive and the percentages therefore exceeded 100% when summed.

#### 3.2.1 Measurement and feature construction of environmental and behavioral indicators

Ten of the 27 included studies (37.0%) used machine learning for measurement and feature construction (T1). These studies constructed environmental and behavioral indicators from image, spatial, survey, and sensor-derived data. Environmental features included streetscape greenery, blue-space exposure, sky visibility, neighborhood characteristics, and other built-environment attributes (6, 7, 16, 22, 62).

The environmental features included streetscape greenery, blue-space exposure, sky visibility, accessibility, neighborhood facilities, and other built-environment attributes. The behavioral and physiological features included travel trajectories, indoor and outdoor movement patterns, mobility regularity, driving characteristics, activity intensity, gait characteristics, and physiological stress responses. These constructed features were subsequently used to examine walking, cycling, public-transport use, driving, loneliness, depression, and stressful older adult–environment interactions.

#### 3.2.2 Prediction of activity behavior, mental health and related health outcomes

Eleven of the 27 included studies (40.7%) used machine learning for outcome prediction or classification (T2), covering behavioral, mental-health, and related health outcomes among older adults. The main mental-health outcomes included depressive symptoms or depression risk, loneliness and social isolation, and psychological well-being, while other predicted outcomes included cognitive impairment, physical function, physical activity, and mobility-related behaviors (31, 32, 58, 59, 63–66). Accordingly, the meaning of “high risk” varied across studies and referred to a higher predicted probability of depression or depressive symptoms, loneliness or social isolation, stressful environmental interactions, or adverse mental- and physical-health outcomes, depending on the outcome modeled.

The predictors integrated in these models included individual and health characteristics, such as age, sex, socioeconomic status, chronic conditions, cognitive status, and prior mental-health indicators; environmental variables, such as population density, land-use mix, street greenery, blue-space exposure, neighborhood facilities, housing conditions, and perceived environmental quality; and behavioral variables, such as walking duration, cycling frequency, public-transport use, driving behavior, physical-activity intensity, travel trajectories, and indoor or outdoor mobility patterns (3–8, 16–23, 29–32, 58–66).

In this context, individualized assessment refers to generating person-specific predicted outcomes or risk scores by jointly considering these environmental, behavioral, and individual-level predictors. It does not necessarily imply clinical diagnosis or treatment recommendation.

#### 3.2.3 Interpretation and association characterization of environment-behavior-health relationships

Twenty-four of the 27 included studies (88.9%) used machine learning for interpretation and association characterization (T3). These analyses served different purposes across environment–activity-behavior and activity-behavior–mental-health relationships. The nine LE–AB studies used random forest, XGBoost, and other gradient-boosting models to examine walking, cycling, public-transport use, driving, and related mobility behaviors (4, 16–23). Frequently examined environmental predictors included population density, land-use configuration, street greenery, neighborhood facilities, accessibility, and perceived environmental quality. Feature-importance measures, partial-dependence plots, SHAP, and GeoShapley were used to characterize predictor importance, nonlinear associations, and possible threshold effects.

The eight AB–MH studies examined the contributions of physical activity, mobility, social participation, health status, and individual characteristics to depression, loneliness, and social isolation (29, 30, 32, 58, 59, 63–65). The four LE–MH studies examined environmental predictors of depression or mental well-being, including air pollution, green and blue space, population density, neighborhood facilities, and housing conditions (5, 6, 8, 60). Across these studies, interpretable models primarily ranked predictor importance and characterized nonlinear or heterogeneous associations rather than establishing causal effects. Two studies (7.4%) used causal-forest or related approaches to examine whether the estimated effects of physical activity or social participation on mental-health outcomes differed across population subgroups (29, 58).

The three T3 studies incorporating variables from all three domains examined how environmental, behavioral, and individual factors jointly contributed to well-being, depression, or stressful older adult–environment interactions (3, 61, 66). Their findings suggested that environmental characteristics were associated with mental-health outcomes in combination with physical activity, social participation, mobility, perceived stress, and other individual-level factors, although the predominance of cross-sectional or small-sample designs limited causal interpretation.

#### 3.2.4 Pathway and mechanism-oriented analysis in environment-behavior-mental-health research

Four of the 27 included studies (14.8%) used machine learning for pathway and mechanism-oriented analysis (T4). Because multi-label coding was applied, these studies could also be classified under measurement, prediction, or interpretation according to the additional functions performed by machine learning.

In these four studies, machine learning was integrated with mediation, structural-equation, or pathway-oriented frameworks to examine potential mechanisms linking living environments and mental-health outcomes through physical activity, perceived stress, social cohesion, environmental perception, and related behavioral factors (7, 8, 58, 62). The analyses therefore used machine learning mainly to identify or characterize potential intermediate pathways rather than to estimate outcomes directly. The reported findings suggested that associations between living environments and mental health may operate through multiple behavioral and psychosocial processes, although the cross-sectional designs and limited number of studies restricted causal interpretation.

Given that only four studies (14.8%) examined pathway or mechanism-oriented questions, evidence in this area remains limited and requires further evaluation using longitudinal designs and stronger causal-inference frameworks.

#### 3.2.5 Causal-effect estimation in environment–behavior–health research

Causal-effect estimation was the least common application of machine learning, identified in two of the 27 included studies (7.4%; T5). Unlike prediction or association characterization, these studies explicitly aimed to estimate the effects of activity behaviors on mental-health outcomes within a causal-inference framework rather than identifying statistical associations alone.

The two studies classified under T5 incorporated machine-learning-assisted causal-inference approaches to examine heterogeneous effects related to mental-health outcomes (29, 58). Takemura et al. (29) combined propensity score matching with causal forest analysis to estimate heterogeneity in the effect of social participation on depressive symptoms among older adults. Zhang et al. (58) integrated causal forest analysis with structural equation modeling and machine-learning models to estimate heterogeneous effects related to depression within a causal-inference framework. Together, these studies represent an emerging application of ML-assisted causal-effect estimation, although their interpretation remains dependent on study design and underlying causal assumptions.

### 3.3 Model validation, interpretation and reporting quality

Model validation and reporting practices varied across the included studies. Among the prediction- and classification-oriented applications, reported performance metrics included AUC-ROC, accuracy, sensitivity, specificity, RMSE, MAE, and pseudo- $R^{2}$ , depending on the outcome and modeling task. However, these metrics were not directly comparable across studies because the studies used different outcomes, data structures, validation designs, and performance measures.

Reported performance values varied across modeling tasks and outcome types. For example, the random-forest model for depression recognition achieved an AUC-ROC of 0.911 and an accuracy of 0.867 (32). In the multimodal stress-detection study, the ensemble bagged-tree model achieved accuracies of 98.13% for stress versus non-stress classification and 98.25% for low- versus high-stress classification (66). For continuous outcomes, the CNN–BiLSTM model reported an $R^{2}$ of 0.867, while XGBoost models achieved $R^{2}$ values ranging from 0.806 to 0.906 (16). In the study of older adults’ walking, random forest achieved an out-of-sample RMSE of 32.15 compared with 39.64 for linear regression (19), whereas the random-forest model for light physical activity reported an MAE of 0.486 and an RMSE of 0.492 (21).

Across the remaining studies, reporting of numerical performance results and validation procedures was inconsistent. Independent external validation was rarely reported, limiting direct comparison of predictive performance across studies.

Model interpretation has become a routine component of machine-learning applications in this field. Rather than reporting predictive performance alone, many studies incorporated interpretable ML techniques, particularly SHAP, feature-importance analysis, GeoShapley, and interaction analysis, to explain model behavior and quantify the contributions of environmental and behavioral variables (8, 16, 19–23, 32, 63–66). These approaches enhanced model transparency and facilitated the interpretation of nonlinear associations and heterogeneous responses identified in Section 3.2.

The completeness of methodological reporting remained heterogeneous. While several studies comprehensively described model development, validation procedures, performance evaluation, and interpretation strategies (8, 58, 63, 65, 66), others reported only the final analytical model or provided limited details regarding parameter tuning, validation design, or model implementation (3, 4, 17–23, 30–32, 61, 66). Overall, the evidence suggests continued progress toward more transparent and interpretable machine-learning applications, although greater consistency in reporting model development and validation would improve the reproducibility and comparability of future studies.

### 3.4 Assessment of risk of bias

The results of the design-specific risk-of-bias and applicability assessments are summarized in Table 3. Consistent with the predefined appraisal frameworks, the JBI and PROBAST+AI assessments are reported separately because they address different study designs and methodological domains.

Among the observational studies assessed using the JBI critical appraisal tools, most studies adequately described their study populations, exposures, outcomes, and analytical methods, although some concerns remained regarding confounding and several design-specific domains. Among prediction-model studies evaluated using PROBAST+AI, concerns were identified most frequently in the analysis and applicability domains, particularly where external validation or reporting of model-development procedures was limited.

Overall, the assessments identified methodological limitations that should be considered when interpreting the evidence, but no additional studies were excluded after the final risk-of-bias appraisal.

## 4 Discussion

### 4.1 Main findings and functional roles of machine learning

Two main patterns emerged from this review. First, the evidence remains fragmented across the relationships among living environments (LE), activity behaviors (AB), and mental health (MH). Most studies addressed individual links among these domains, whereas relatively few incorporated LE, AB, and MH within the same analytical framework. Current evidence therefore provides a stronger understanding of specific relationships than of how environmental, behavioral, and mental-health dimensions are interconnected as a whole.

Second, as summarized across the evidence-building process in Figure 3, the functional roles of machine learning were unevenly distributed across the T1–T5 framework. Current applications were concentrated primarily in measurement and feature construction, prediction, and association characterization, whereas pathway-oriented and causal-effect applications were less common. This distribution indicates that ML has so far contributed mainly to extending the measurement and modeling of complex environment–behavior–mental-health relationships, while its application to pathway-oriented and effect-oriented questions remains comparatively limited.

**Figure 3:**
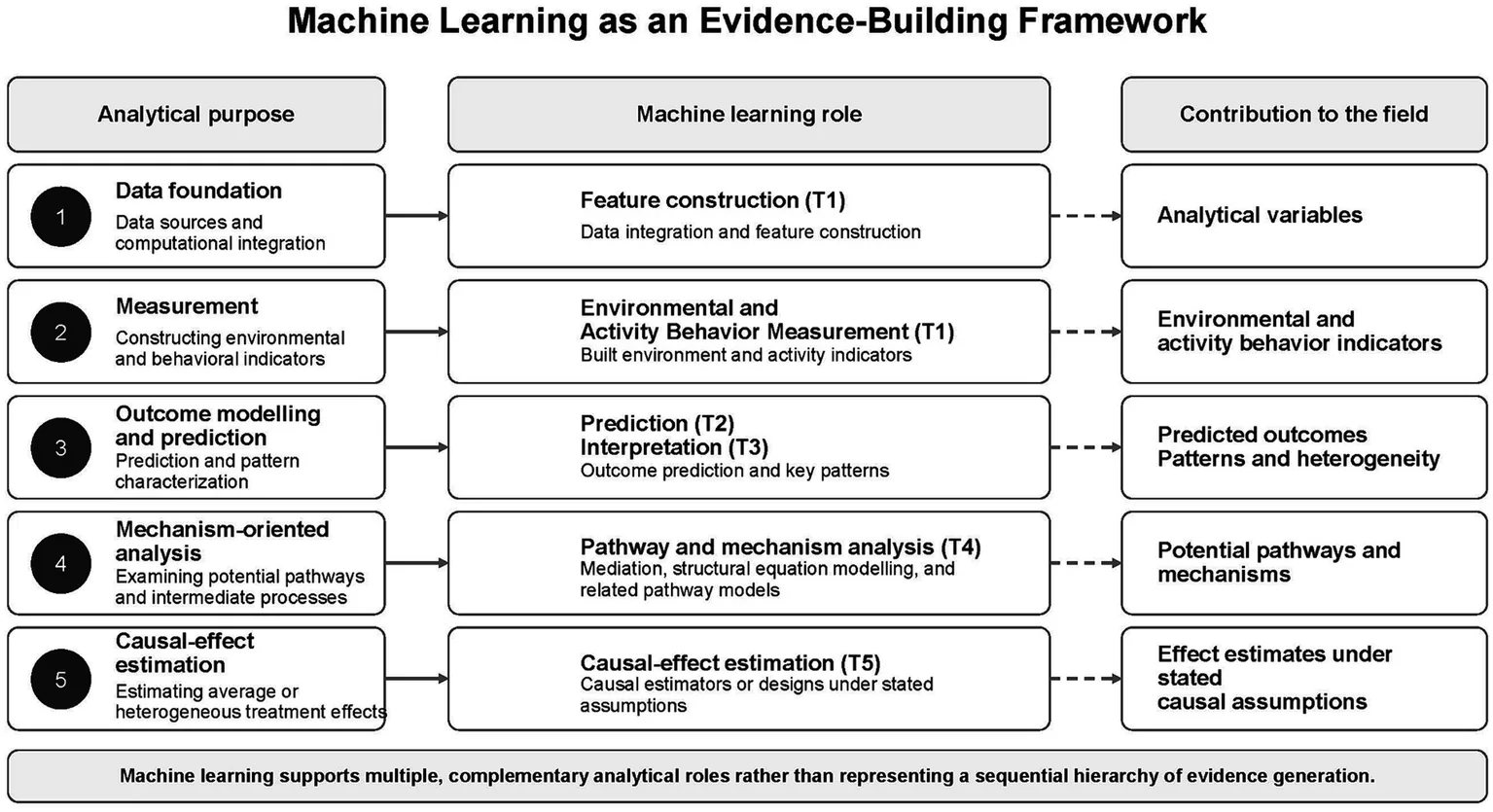
Machine learning roles across the evidence-building process for healthy and active aging.

### 4.2 Evidence heterogeneity, generalizability, and future research priorities

The evidence synthesized in this review was characterized by substantial heterogeneity in study design, population, geographic context, data source, and the measurement of living environments, activity behaviors, and mental health. Much of the literature relied on cross-sectional or secondary data, while environmental and behavioral exposures were derived from diverse sources, including surveys, spatial data, street-view imagery, mobility records, and sensor-based measures. Such variation expands the range of relationships that can be examined but also limits direct comparison across studies, because differences in observed patterns may reflect not only underlying environment–behavior–health relationships but also differences in sampling, measurement, and analytical context.

Generalizability therefore remains an important challenge. Models developed within a specific population, geographic setting, or data-collection system may not reproduce the same relationships when applied elsewhere, particularly when environmental characteristics, behavioral patterns, or measurement procedures differ. Although internal validation was commonly used in prediction-oriented studies, independent external or spatial validation was less frequently reported. Consequently, the transferability of many ML-derived patterns across populations and places remains uncertain.

Future research should address these limitations through stronger study designs and more systematic validation. Longitudinal and repeated-measure studies would improve the temporal characterization of environmental, behavioral, and mental-health changes, while multisite studies could provide a stronger basis for assessing variation across geographic and population contexts. Greater use of independent external and spatial validation would further clarify whether identified patterns are robust beyond the datasets in which they were developed. More consistent definitions, measurement procedures, and reporting of environmental exposures, activity behaviors, mental-health outcomes, and validation strategies would also facilitate cross-study comparison and cumulative evidence synthesis.

### 4.3 Model interpretation, pathway analysis and causal boundaries

An important distinction emerging from this review is that association characterization, pathway analysis, and causal inference represent different levels of explanation and should not be treated as interchangeable. Identifying a strong, nonlinear, or heterogeneous association can improve understanding of how environmental, behavioral, and mental-health variables are related, but it does not by itself establish the process through which that relationship occurs. Similarly, identifying a plausible pathway between variables can support hypotheses about how different domains may be connected, but evidence of a pathway does not necessarily establish a causal effect. Maintaining these distinctions is particularly important in a literature where prediction, interpretation, pathway analysis, and causal-effect estimation increasingly coexist within related analytical workflows.

The evidential contribution of ML therefore depends on the analytical question it is used to address and the framework within which it is embedded. ML can strengthen the characterization of complex relationships and can support pathway-oriented or effect-oriented analyses, but the use of ML does not itself transform associational evidence into mechanistic or causal evidence. Greater model complexity, predictive performance, or interpretability should likewise not be taken as indicating stronger causal inference.

Accordingly, the strength of evidence concerning mechanisms or causal effects depends primarily on the underlying study design, the assumptions required for inference, and the analytical framework used to connect the data to the research question. This distinction provides an important basis for interpreting the expanding role of ML in research on living environments, activity behaviors, and mental health: methodological sophistication should be evaluated not only by what a model can detect or predict, but also by whether the study design and inferential framework support the conclusions being drawn from it.

### 4.4 Practical implications

The reviewed applications of ML offer several potential contributions to research-informed age-friendly planning. Fine-grained environmental and behavioral measures derived from spatial, image, mobility, and sensor data can provide more detailed information on how older adults experience and interact with their living environments. Interpretable ML models can further identify spatial variation, influential environmental and behavioral factors, and population heterogeneity associated with activity behaviors and mental-health outcomes. These capabilities may complement conventional environmental assessment by providing more context-sensitive information for identifying areas and population groups with different environmental and behavioral characteristics.

At present, the practical contribution of these approaches lies primarily in supporting evidence generation and exploratory assessment. Their use in planning and public-health decision-making requires consideration of local context, population characteristics, data availability, and model transferability. Evidence directly evaluating implementation, intervention outcomes, and decision utility remains limited, leaving the translation of ML-derived findings into planning actions an important area for further evaluation. Future research should therefore move beyond exploratory evidence generation toward evaluating whether ML-informed insights improve real-world decision utility and intervention outcomes in planning and public-health contexts.

### 4.5 Strengths and limitations of the review

This review provides a structured synthesis of the functional roles of machine learning across research on living environments, activity behaviors, and mental health among older adults. The eligibility criteria and explicit operationalization of activity behaviors improved the conceptual consistency of study classification, while the multi-label T1–T5 framework enabled studies to be characterized according to the different analytical functions performed by ML. Eligibility decisions, data extraction, task classification, and risk-of-bias assessments were checked by a second reviewer to improve consistency.

Several limitations should nevertheless be acknowledged. First, the review was not prospectively registered. Second, substantial heterogeneity in study designs, measurements, ML applications, and reported outcomes precluded quantitative meta-analysis. Accordingly, the review focused on structured comparative and narrative synthesis rather than pooled effect estimation. Third, data extraction and risk-of-bias assessment were initially conducted by one reviewer and checked by a second reviewer rather than completed independently in duplicate; therefore, some reviewer-dependent judgement may remain.

## 5 Conclusion

This review shows that machine learning is being applied across multiple, partially connected relationships among living environments, activity behaviors, and mental health in older adults. Its applications extend from environmental and behavioral measurement to prediction and the characterization of nonlinear, interactive, and heterogeneous relationships.

Current evidence remains concentrated on individual links among these domains, with relatively limited integration of living environments, activity behaviors, and mental health within the same analytical framework. Greater integration of environmental, behavioral, and mental-health evidence across populations, places, and time could strengthen understanding of how these domains are interconnected in later life.

## Data Availability

The original contributions presented in this study are included in the article and its tables. Further inquiries can be directed to the corresponding author.
